# Enhancing Recruitment of Adolescents Aged 16-18 Years in a Web-Based Peer Network Study Through Financial Reimbursements: Randomized Controlled Trial

**DOI:** 10.2196/70223

**Published:** 2025-11-25

**Authors:** Sarah Eddy, Rachel Sacks-Davis, Michelle Raggatt, Cassandra Wright, Paul Dietze, Margaret Hellard, Jane Hocking, Megan S C Lim

**Affiliations:** 1 Burnet Institute, 85 Commercial Rd, Melbourne, 3004, Australia, 61 0385062403; 2 School of Population Health and Preventive Medicine, Monash University, Melbourne, Australia; 3 School of Population and Global Health, University of Melbourne, Melbourne, Australia; 4 Menzies School of Health Research, Darwin, Australia; 5 National Drug Research Institute, Curtin University, Perth, Australia; 6 Alfred Health, Melbourne, Australia

**Keywords:** financial reimbursement, incentive, adolescent, peer, young person, web-based respondent-driven sampling, peer network, social network

## Abstract

**Background:**

Peers are known to influence the health behaviors and attitudes of adolescents, yet recruitment of these networks is challenging. Previous studies have used web-based respondent-driven sampling methods to recruit this population, yet none have experimentally investigated the impact of financial reimbursements.

**Objective:**

This study aimed to (1) compare the effectiveness of two financial reimbursement strategies for recruiting adolescents and their peer networks and (2) explore factors associated with successfully recruiting peers.

**Methods:**

A parallel-design randomized controlled trial was conducted in which participants (seeds) were randomly allocated to a fixed cash reimbursement (control) or scaled reimbursement (experimental) group as a strategy to be recruited into a web-based peer network study. Seeds aged 16 to 18 years were recruited through social media advertisements and an online student panel. They completed a web-based survey, which assessed eligibility and included questions about their friends (peers). Allocation occurred through a survey platform using a simple randomization method. In the fixed group, all participants in a peer network received AUD $5 (US $3.29); in the scaled group, all participants in a peer network received an additional AUD $5 (US $3.29) per peer who successfully completed the survey (up to AUD $30 each [US $19.72]). Participants and researchers were not blinded to intervention groups. The primary outcome was recruitment of peers to complete the web-based survey (proportion of nominated peers). The number of peers recruited was a secondary outcome. In secondary analyses, we identified peer-, relationship-, and seed-level variables associated with successfully recruiting peers.

**Results:**

Of 463 seeds allocated to an intervention (scaled n=221 and fixed n=242), 319 (68.9%) had complete data for analysis (scaled n=157, 71% and fixed n=162, 67%). A total of 11.9% of seeds successfully referred peers (18.5% scaled group and 5.6% fixed group). Those in the scaled reimbursement intervention were 3.80 times more likely to successfully recruit their peers than those in the fixed reimbursement intervention (proportion ratio 3.80, 95% CI 1.78‐8.09). Similarly, the average number of peers recruited differed by 0.19 (95% CI 0.11‐0.28) per seed between the scaled and fixed intervention groups. Peer recruitment success was similar regardless of the gender, age, education level, and network size of seeds or the gender, age, and closeness of peers. Seeds recruited through social media were more likely to successfully recruit their nominated peers than those recruited through a research panel (proportion ratio 2.20, 95% CI 1.06‐4.55).

**Conclusions:**

Scaled reimbursements resulted in significantly greater recruitment of peers than fixed reimbursements; however, the total number of peers recruited was low. Greater-value incentives and stronger initial recruitment through social media may be needed to recruit large numbers of friend networks.

## Introduction

### Background

The period from adolescence to adulthood is a significant period of transition during which key health risk behaviors emerge. This time is characterized by experimentation with alcohol and other drugs and sexual behaviors and the onset of mental health issues [1]. Researchers have consistently highlighted the importance of the social context in understanding the development of both positive and negative adolescent health behaviors [2,3]. As emphasized in social network theory, an individual’s social network influences their attitudes and behaviors [3]. Peer influence is thought to occur through different mechanisms, including homophilic social selection—the selection of friends based on shared behaviors or attitudes—and social influence—the convergence of similar behaviors and attitudes within a network over time [4]. The presence of peers can increase adolescent susceptibility to risky behaviors, including sexual risk taking and alcohol use [5]. There is extensive evidence of high similarity in health behaviors among networks of young people [4,6-8]. By studying adolescents in the context of their peer networks, health interventions can be designed that utilize these social networks.

To date, studies investigating adolescent social networks have examined health behaviors such as smoking, alcohol and substance use, physical activity, and dieting [4]. However, these studies have predominantly taken place in school settings, using an existing dataset (1994‐2002) from the United States [4]. While it is convenient and useful to sample adolescents and their friendships within schools, this approach does not reach those disengaged from mainstream education or capture the transitory period after completing school. Less is known about the influence of nonschool peer relationships, particularly relevant in the current social media era where relationship boundaries are less defined. On social media, data are inherently organized in a network and provide an opportunity to study these processes at a larger scale [9].

Respondent-driven sampling (RDS) is a recruitment method typically used for hard-to-reach populations, where *seeds* (primary participants) are recruited and then asked to recruit several *peers* (secondary participants) within their network [10]. In recent years, there has been an increase in the use of web-based RDS (WebRDS), an online version of RDS that allows participants to be recruited without the need for face-to-face contact [11]. This method confers significant advantages over traditional RDS, including ease of access, reductions in time and cost, and respondent privacy and anonymity [10,12]. Equally, researchers have identified drawbacks of the web-based approach, including the increased potential for fraudulent and duplicate responses, lower quality data, and key differences in the population recruited (eg, higher socioeconomic status, fewer Aboriginal and Torres Strait Islander participants, and more participants who speak a language other than English at home) [10,12]. Despite these challenges, the nature of WebRDS provides an opportunity to study the interactions between and within networks in an online format. Existing studies have recruited seeds through social media ads (eg, Facebook), through online research panels, or through the researcher’s networks and often include some researcher contact (ie, via phone or email) to confirm eligibility or motivation to engage [11]. Seeds typically recruit their peers by sharing a link via direct messaging (Facebook or WhatsApp) or email (see Helms et al [11] for a review). Since young people’s lives largely exist online, and those engaging in higher risk behaviors are difficult to reach through traditional recruitment methods, RDS is well suited to recruiting young people and their peer networks.

While recruitment of peer networks can be challenging, one factor typically associated with successful recruitment is financial reimbursement, whereby seeds receive additional reimbursement for each peer they recruit [11]. In WebRDS studies conducted with adolescent and youth samples, seed reimbursement has ranged from US $4 to 30, with an additional incentive of US $4 to 20 per peer successfully recruited (maximum 2-5 referrals) [12-16]. Many studies have shown that offering incentives significantly improves individual participation in research [17]. However, to our knowledge, no studies to date have compared the effectiveness of different incentives for recruiting peers among adolescents and young people. One study investigated the best way to use internet-based RDS to recruit friends into an existing research panel within the general population and found that the combination of higher incentives for each peer recruited and the inclusion of a sign-up bonus (for joining the research panel) was effective at increasing the number of friends recruited [18]. The preference for incremental incentives (ie, an additional reimbursement for each peer) over a fixed amount (regardless of the number of peers recruited) was also emphasized in an adult sample of men who have sex with men [19]. Notably, this study reported that monetary incentives were more important for younger participants [19]. It remains unclear how varying financial reimbursements affect a young person’s willingness to refer peers.

Demographic, relational, and methodological factors may also have an impact on recruitment success. While often seeds are more likely to recruit peers with similar demographic characteristics due to homophilic selection, this does not always explain recruitment differences [20]. Some studies within the general population have shown that productive seeds (in terms of recruitment or referrals) are more likely to be younger, female, more highly educated, and with a larger peer network [21]. Online recruitment and a smaller number of initial seeds have also been identified as key factors [11]. Further research within a youth sample is needed to understand the characteristics of successful seeds and their referred peers.

### Rationale

We conducted a randomized controlled trial that aimed to determine the effectiveness of using WebRDS to recruit adolescent peer networks. Importantly, this study addresses a gap in the literature by investigating the impact of two different reimbursement structures on recruitment success—a scaled reimbursement (experimental) group, in which all participants in a peer network received an additional AUD $5 (US $3.29) for each peer who was successfully recruited, to a maximum value of AUD $30 each (US $19.72); and a fixed reimbursement (control) group, in which all participants were reimbursed AUD $5 (US $3.29) for completing the survey. In the context of ongoing challenges with recruiting peer networks, this study provides valuable information to determine the feasibility and cost of monetary incentives in future WebRDS studies.

### Aims and Research Questions

The primary aim was to compare the effectiveness of two financial reimbursement strategies for recruiting peer networks, explored through the following research questions:

1. Does a scaled group financial reimbursement for peer referral increase the proportion of an individual’s peer network who complete the survey compared to having no peer referral reimbursement?
2. Does a scaled group financial reimbursement for peer referral increase the number of peers per participant who complete the survey compared to having no peer referral reimbursement?

We hypothesized that a scaled group financial reimbursement for peer referral would result in a significantly higher proportion of participants’ peer networks and a significantly higher number of peers per participant who complete the survey, compared to a fixed reimbursement rate for individuals.

A secondary aim was to determine what demographic, relational, and methodological factors were associated with successfully recruiting peers. As this was an exploratory analysis, no hypotheses were specified.

## Methods

### Study Design

To test the effectiveness of reimbursements for peer referral in a web-based cross-sectional peer network study, a 2-group parallel randomized controlled trial (RCT) design was used. Participants were then randomly allocated in a 1:1 ratio to the scaled group reimbursement (experimental) or the fixed group reimbursement (control). The study design was informed by qualitative interviews with young people [22]. This RCT was not registered as the primary outcome (recruitment of friends) was not a direct health or medical outcome. The trial is reported in accordance with the CONSORT-EHEALTH (Consolidated Standards of Reporting Trials of Electronic and Mobile Health Applications and Online Telehealth) guidelines for web-based RCTs [23]. Further details can be found in the published protocol [22]. A deviation from this published protocol occurred due to lack of success recruiting participants via social media. Recruitment was slower than anticipated, and a greater number of participants were recruited from Student Edge instead of social media (see Recruitment section).

### Participants

In this study, seeds were defined as individuals recruited directly through online recruitment platforms who had the opportunity to recruit their friends. Peers were defined as friends nominated by seeds to complete the survey. Seeds were eligible if they were aged between 16 and 18 years and currently living in Australia; peers were eligible if they were aged 16 years and older and currently living in Australia. A peer network encompassed a seed and their peers.

### Recruitment

Recruitment initially targeted participants through three platforms: Instagram or Facebook, Snapchat, and Student Edge panel (detailed further in protocol [22]). Instagram and Facebook were treated as a single recruitment source, given that they are managed through the same advertising account and owned by the parent company Meta. Student Edge is a web-based organization that hosts online surveys targeted at young people in high school and technical and further education in Australia [24]. The survey was made available to Student Edge members on their website, while recruitment through social media comprised paid advertisements. Advertisements in still and video format were designed to appeal to young people, and they only briefly mentioned the study topic and the reimbursement. Recruitment initially took place from March 21 to April 2, 2022. Initial recruitment through social media platforms posed challenges, with no participants successfully recruited through Snapchat and only small numbers recruited through Instagram or Facebook. As such, in early 2023, the advertisements were revised into video format with brighter colors and more dynamic text and hosted on TikTok as well as Instagram or Facebook, with the goal of increasing recruitment through social media platforms. TikTok is a free hosting platform for short-form videos, primarily accessed via smartphone. As was the case with Snapchat, TikTok advertising proved unsuccessful, while a small additional sample of young people was recruited through Instagram or Facebook with the revised advertisements. Following this, the remainder of the target sample was recruited through the Student Edge platform. Student Edge participants who completed the survey in 2022 were unable to complete it a second time. This second phase of recruitment occurred between March 15 and May 12, 2023. In total, across both years, active recruitment took 10 days more than anticipated.

### Intervention

First, seed participants answered questions about their own behaviors and then were asked to specify their number of “close friends” (peers), up to a maximum of 10. Seed participants were then asked to answer questions about each peer, including initials and a chosen codename, and advised of the reimbursement amount depending on their reimbursement group. At the completion of the survey, seeds had the opportunity to either personalize a message or use a suggested message inviting their peers to participate in the survey. Seeds received this information in an email immediately after completing the survey and again after 72 hours. Seed participants were provided with a unique link via email to share with their peers, which included their record ID. Peers were automatically recruited in the same reimbursement group as their seed. When commencing the survey, peers were asked to provide their given codename and their initials. Peer networks were linked using the seed’s ID number and peer initials and codenames. Linkage required a match of seed ID and at least one of the peer’s codename or initials. Peers were also asked questions about their own peer network, yet were not able to refer them to complete the survey.

All seeds and peers in the fixed reimbursement group received AUD $5 (US $3.29) for completing the survey, with no additional reimbursement for peers in their network who successfully completed the survey. In the scaled reimbursement group, seeds and peers within a peer network received AUD $5 (US $3.29) for completing the survey, plus an additional AUD $5 (US $3.29) for each peer who successfully completed the survey, up to a maximum of AUD $30 (US $19.72) per participant (detailed in [22]). At the completion of the recruitment phase, participants were reimbursed with online gift vouchers sent via SMS text messaging through a third-party service. Due to the existing platform features, participants recruited through Student Edge received the first AUD $5 (US $3.29) through the Student Edge platform and any additional value in the form of gift vouchers. Participants could contact the research team via an online form if they encountered any issues with the survey.

### Outcomes

The primary outcome was defined as completion of the survey by a peer (yes/no). We estimated the difference in the proportion of seeds’ nominated peers who completed the survey in the scaled group reimbursement compared to the fixed individual reimbursement group. Survey completion was recorded if the peer clicked the final survey submission button. This outcome measure indicates the level of complete data about each seed’s peer network.

The secondary outcome was the number of a seed’s peers who completed the survey. We estimated the difference in the number of peers who completed the survey per seed in the scaled group reimbursement compared to the fixed individual reimbursement group. This outcome measure assesses whether the reimbursement model results in an overall higher number of participants.

### Randomization

Randomization (generation and allocation) occurred automatically through the REDCap survey platform [25]. This involved a simple randomization based on the final digit (seconds) of the main survey page timestamp. Those who commenced the survey with an even number of seconds were allocated to the scaled (experimental) group, and those with an odd number of seconds were allocated to the fixed (control) group. The intervention was identical, differing only in the amount of reimbursement offered. Randomization occurred after consent, yet participants were only advised of which intervention group they had been allocated to at the end of the main survey section, prior to answering questions about their peers. Researchers were not blinded to allocation on the survey platform yet did not interact with participants directly. Participants were unaware of the other experimental condition.

### Data Collection

Participants completed an online survey through REDCap. Participants could not proceed with the survey if they did not meet the age criteria. The survey captured potential factors associated with successfully recruiting peers. This included seed-level factors such as demographic characteristics, recruitment setting, and network size. Peer-level variables included perceived closeness, age, and gender. To increase engagement, seeds could choose a codename and avatar to identify themselves and a codename and avatar to identify each peer. The survey also covered topics such as social media and media related to sexual health (ie, pornography, sexting, and dating apps), mental health, sexual health, alcohol and other drug use, and COVID-19 vaccination, which will be reported on elsewhere.

### Sample Size and Statistical Analysis

We determined the required sample size of seeds to detect a difference in the proportion of an individual’s peer network that completed the survey between each intervention group. In line with previous research, we predicted that seeds would refer an average of three peers [26,27] and that approximately half of participants in the scaled group would successfully recruit at least one peer (as indicated by the peer’s completion of the survey) [12,15]. Assuming the probability of recruiting each peer is binomial and the probability of recruiting no peers being (1−*p*)*^n^* (where *n* is the total number of peers and *p* is the probability of any one nomination being recruited), the probability of any nomination completing the survey was 0.2. We assumed some variation between friendship groups, including an intracluster correlation of 0.5 and a mean cluster size of 3, with a coefficient of variation for cluster size of 0.83. On this basis, 153 seeds were required per intervention group (total of 306 participants). This estimate allowed us to detect a difference of 1.4 peers per seed completing the survey between intervention groups, with 80% power. Conservatively, we estimated a total of 92 peers across both groups. We intended to recruit equal numbers from each of the three recruitment settings [22], yet due to the limited success with social media advertising outlined above, a greater number of participants were recruited through the Student Edge platform.

To assess balance in demographic characteristics between seeds in the two intervention groups, descriptive statistics were reported by intervention group in table form, reporting mean and SD for continuous data and number of participants (n) and percentages for categorical data.

The primary outcome was evaluated in a modified intention-to-treat analysis where participants were excluded from analysis if they did not answer questions about their peers, as this was considered an incomplete response. The exception to this was if participants were identified as having zero close friends. To assess the effect of the reimbursement intervention on the proportion of a seed’s peers who completed the survey, we used generalized estimating equations (GEE) with a log link, binomial family, and exchangeable correlation structure to account for within friendship group correlation. In this analysis, peer nominees were the unit of analysis, grouped by their seed. Each peer could either be successfully recruited or not recruited (binary outcome). This analyzed seeds who nominated at least one peer.

To determine the effect of the intervention on the number of peers per participant who completed the survey (secondary outcome), we estimated the expected difference in counts between groups using predictions from a Poisson regression model. Poisson regression was chosen for analysis based on the distribution of the number of peers. In secondary analyses, we identified factors associated with successfully recruiting each peer, considering peer-, relationship-, and seed-level variables. Predictor selection was informed by prior studies investigating online survey recruitment [11,20,21]. We used bivariable log binomial GEE, where the outcome was the successful recruitment of a nominated peer. Potential predictors included recruitment setting, demographics, perceived closeness, and network size. Exchangeable correlation structures were used to account for clustering. In addition, we identified factors associated with recruiting a larger number of peers. We used bivariable Poisson regression models. Only seed-level variables were considered for the latter analysis. For simplicity in interpretation, demographic variables in secondary analyses were included in categorical form or converted into categories, and “I don’t wish to say” responses were coded as missing.

### Ethical Considerations

This study received approval from the Monash University Human Research Ethics Committee (project 23132). Participants were provided with information about the study in both video and written form. Informed consent was obtained through a series of checkboxes, indicating that participants had understood the information provided and were happy to take part and share their information for the study purpose. All data were deidentified for analysis. As described in detail earlier in the Intervention section, participants received between AUD $5 (US $3.29) and AUD $30 (US $19.72) in compensation. Details of relevant support services were provided at the completion of the survey.

## Results

### Overview

**Figure 1.**
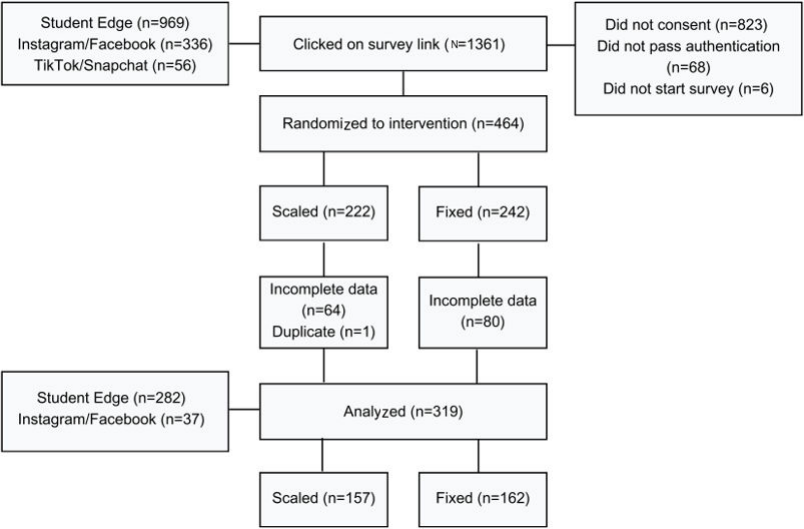
Flowchart showing seed involvement in the intervention from recruitment through to analysis.

A total of 1361 seeds clicked on the survey link. Of these, a total of 897 did not provide consent, pass authentication (due to being identified as a duplicate), or commence the main survey following authentication (see Figure 1). The remaining 464 participants were randomized to the scaled intervention (n=222) or the fixed intervention (n=242). Of these, 144 only partially completed the survey, and 1 was identified as a duplicate. Thus, 319 participants were analyzed (n=157 in the scaled group and n=162 in the fixed group). Of those with partial completion, 127 stopped the survey post unblinding. Post-unblinding attrition was higher in the fixed group (n=74, 30.6%) than in the scaled group (n=53, 23.9%).

The demographic characteristics of seeds overall and separated by intervention group are shown in Table 1. Intervention groups were balanced in demographic characteristics. Overall, the sample had a mean age of 17.03 (SD 0.8) years, and 60.8% (n=194) identified as a woman. Most were from New South Wales (n=128, 40.1%) or Victoria (n=71, 22.3%), were currently studying (n=299, 66.2%), and spoke English at home (n=194, 60.8%). Most of the sample (n=282, 88.4%) were recruited through the Student Edge panel, and almost two-thirds (n=198, 62.1%) were recruited during the second wave of data collection in 2023, yet these characteristics were consistent across intervention groups. There were no missing data as participants were required to answer each question, with the option to select “I don’t wish to say.” Of variables included in secondary analyses, seed-level gender (n=4, 1.3%) and education level (n=9, 2.8%), and peer-level gender (n=7, 2.3%) and closeness (n=1, 0.5%), contained a small number of “I don’t wish to say” responses, which were not included in the analysis.

**Table 1.** Demographic characteristics of primary participants.

Variables	Scaled (n=157)	Fixed (n=162)	Total (n=319)
Age (years), mean (SD)	17.03 (0.8)	17.04 (0.8)	17.03 (0.8)
Gender, n (%)			
Man	49 (31.2)	54 (33.3)	103 (32.3)
Woman	99 (63.1)	95 (58.7)	194 (60.8)
Nonbinary	7 (4.4)	11 (6.8)	18 (5.6)
I don’t wish to say	2 (1.3)	2 (1.2)	4 (1.3)
State, n (%)			
Australian Capital Territory	1 (0.6)	2 (1.2)	3 (0.9)
New South Wales	63 (40.1)	65 (40.1)	128 (40.1)
Northern Territory	0 (0)	1 (0.6)	1 (0.3)
Queensland	17 (10.8)	13 (8)	30 (9.4)
South Australia	10 (6.4)	10 (6.2)	20 (6.3)
Tasmania	1 (0.6)	3 (1.9)	4 (1.2)
Victoria	35 (22.3)	36 (22.2)	71 (22.3)
Western Australia	29 (18.5)	27 (16.7)	56 (17.6)
I don’t wish to say	1 (0.7)	5 (3.1)	6 (1.9)
Remoteness, n (%)			
Nonmajor city	12 (7.7)	13 (8)	25 (7.8)
Major city	117 (74.5)	120 (74.1)	237 (74.3)
I don’t wish to say	28 (17.8)	29 (17.9)	57 (17.9)
Language other than English spoken at home, n (%)			
No	99 (63.1)	95 (58.6)	194 (60.8)
Yes	58 (36.9)	61 (37.7)	119 (37.3)
I don’t wish to say	0 (0)	6 (3.7)	6 (1.9)
Aboriginal or Torres Strait Islander, n (%)			
No	152 (96.8)	153 (94.4)	305 (95.6)
Yes	2 (1.3)	4 (2.5)	6 (1.9)
I don’t wish to say	3 (1.9)	5 (3.1)	8 (2.5)
Currently studying, n (%)			
No	3 (1.9)	9 (5.5)	12 (3.8)
Yes	151 (96.2)	148 (91.4)	299 (93.7)
I don’t wish to say	3 (1.9)	5 (3.1)	8 (2.5)
Current or highest level of study, n (%)			
High school	104 (66.2)	107 (66.1)	211 (66.2)
Post high school	50 (31.9)	49 (30.2)	99 (31)
I don’t wish to say	3 (1.9)	6 (3.7)	9 (2.8)
School type, n (%)			
Public	108 (68.8)	102 (63)	210 (65.8)
Catholic	17 (10.8)	28 (17.3)	45 (14.1)
Private	31 (19.8)	26 (16.1)	57 (17.9)
I don’t wish to say	1 (0.6)	6 (3.7)	7 (2.2)
Sexual identity, n (%)			
Heterosexual	103 (65.6)	106 (65.5)	209 (65.5)
LGBTQIA^a^	53 (33.8)	54 (33.3)	107 (33.5)
I don’t wish to say	1 (0.6)	2 (1.2)	3 (1.0)
Recruitment setting, n (%)			
Instagram/Facebook	17 (10.8)	20 (12.4)	37 (11.6)
Student Edge	140 (89.2)	142 (87.6)	282 (88.4)
Recruitment year, n (%)			
2022	63 (40.1)	58 (35.8)	121 (37.9)
2023	94 (59.9)	104 (64.2)	198 (62.1)

a LGBTQIA: lesbian, gay, bisexual, transgender, queer, intersex, and asexual.

### Primary Analyses

#### Overview

Participants nominated an average of 3.47 (IQR 2‐5, range 0‐10) peers. Peers who were ineligible based on age were excluded (n=4). This left 298 (of 319) participants who had nominated at least one eligible peer. Of 1069 total peers nominated, 55 completed the survey, of which 48 (aged 16-21 y) were successfully linked with their seed. The remaining peers were unable to be linked via the ID number of their referring seed and peer initials or codename. In total, 11.9% (n=38) of seeds successfully recruited a peer (18.5% scaled and 5.6% fixed). Only participants in the scaled group recruited more than one peer (3.8% recruited two peers and 1.3% recruited three peers).

#### Primary Outcome

Those in the scaled reimbursement group were approximately 3.80 times more likely to successfully recruit their nominated peers than those in the fixed reimbursement group (proportion ratio 3.80, 95% CI 1.78‐8.09).

#### Secondary Outcome

Results from the Poisson regression analysis showed an effect of intervention group on the number of peers recruited. Each participant in the scaled group recruited an average of 0.25 peers, while those in the fixed group recruited an average of 0.06 peers, resulting in a difference of 0.19 (95% CI 0.11‐0.28) between groups.

### Secondary Analyses

#### Primary Outcome

Results from exploratory bivariable log binomial GEE analyses can be found in Table 2. The proportion of nominated peers successfully recruited was similar regardless of the gender, age, education level, and network size of seeds, or the gender, age, and closeness of peers. On average, seeds recruited through Instagram or Facebook were approximately two times as likely to successfully recruit their nominated peers. However, the CI was wide, indicating the difference between seeds recruited through Instagram or Facebook and those recruited through Student Edge was relatively imprecise (proportion ratio 2.20, 95% CI 1.06‐4.55).

**Table 2.** Seed and peer-level factors associated with successful recruitment of a nominated peer.

Variables	PR^a^ (95% CI)
Seed level	
Gender	
Man	—^b^
Woman	1.04 (0.52-2.09)
Nonbinary	1.82 (0.61-5.31)
Age (years)	
16	—
17	0.83 (0.37-1.85)
18	1.02 (0.48-2.18)
Education	
High school	—
Post high school	1.05 (0.54-2.06)
Recruitment method	
Student Edge	—
Instagram/Facebook	2.20 (1.06-4.55)
Network size	
≤5 peers	—
>5 peers	0.46 (0.17-1.21)
Peer level	
Gender	
Man	—
Woman	0.89 (0.48-1.67)
Nonbinary	1.93 (0.75-4.97)
Age (years)	
16	—
17	0.56 (0.28-1.13)
18	0.50 (0.22-1.15)
≥19	0.98 (0.41-2.38)
Closeness	
Not at all/slightly/moderately	—
Very/extremely	1.13 (0.62-2.05)

a PR: proportion ratio.

b Not applicable.

#### Secondary Outcome

Results from bivariable Poisson regression analyses using seed-level variables can be found in Table 3. The number of nominated peers successfully recruited was similar regardless of the gender, age, education level, and network size of seeds. On average, seeds recruited through Instagram or Facebook were likely to recruit approximately two and a half times more peers. However, the wide CI indicates that the estimate of this difference between seeds recruited through Instagram or Facebook and those recruited through Student Edge was relatively imprecise (count ratio 2.54, 95% CI 1.32‐4.88).

**Table 3.** Seed-level factors associated with recruiting a larger number of nominated peers.

Variables	CR^a^ (95% CI)
Gender	
Man	—^b^
Woman	0.99 (0.53-1.86)
Nonbinary	1.91 (0.69-5.25)
Age (years)	
16	—
17	0.93 (0.45-1.89)
18	1.08 (0.54-2.15)
Educated	
High school	—
Post high school	0.97 (0.53-1.78)
Recruitment method	
Student Edge	—
Instagram/Facebook	2.54 (1.32-4.88)
Network size	
≤5 peers	—
>5 peers	1.13 (0.53-2.41)

a CR: count ratio.

b Not applicable.

### Reimbursement Costs

Excluding additional costs associated with using the student panel or advertising the study on social media, overall, the average reimbursement per participant was AUD $6.39 (US $4.20). Separated by group, average reimbursement per participant was AUD $5 (US $3.30) in the fixed intervention and AUD $7.60 (US $5.00) in the scaled condition. Specifically, an average seed received AUD $5.61 (US $3.68; fixed group AUD $5, US $3.29; scaled group AUD $6.24, US $4.10), and an average peer received AUD $11.56 (US $7.60; fixed group AUD $5, US $3.29; scaled group AUD $13.08, US $8.60).

## Discussion

### Principal Results

As predicted, a scaled reimbursement strategy that included additional incentives for each peer in a network was more effective than a fixed reimbursement strategy in recruiting adolescent peer networks. Those in the scaled reimbursement group recruited a greater proportion of nominated peers and recruited a greater average number of peers, compared to the fixed reimbursement group. Additionally, seeds recruited through Instagram and Facebook were more likely to recruit peers and each recruit a higher number of peers than those recruited through Student Edge. Other demographic and relational factors of seeds or peers were not found to have an impact on peer recruitment success.

### Comparison With Prior Work

While we are not aware of any studies to date that have compared the relative effectiveness of different financial incentives for recruiting adolescent peers, our results are consistent with those from a WebRDS study with a sample of men who have sex with men, which identified a preference for scaled over fixed reimbursement for recruiting peers in their network [19]. More broadly, they also reflect the findings of a review that reported lower recruitment success among WebRDS studies with no monetary incentive [11]. The study by Schonlau et al [18] is also relevant, as it experimentally varied reimbursement amounts for each peer recruited (US $15 vs $30) and the inclusion of sign-up bonuses for peers (US $0 vs $20) to determine the most effective way to recruit adults into an internet panel. The combination of higher incentives and a sign-up bonus significantly increased peer recruitment compared to either one alone [18]. Despite key differences between that study and the current study, this highlights the importance of adequate monetary reimbursements for both seeds and peers. In our study, the additional cost for scaled reimbursements was AUD $2.60 (US $1.68) per participant, which is minimal when considered against the total expenses of conducting the study. Although peer recruitment in the scaled condition was improved compared to the fixed condition (18.5% vs 5.6%), overall peer recruitment in this study was low (11.9%). We conservatively estimated recruiting 92 peers, yet only obtained 55, not all of whom could be linked automatically to their seed due to ethical constraints. Despite allowing participants to recruit up to 10 peers each and incentives scaling up to the fifth peer for those in the scaled group, very few seeds recruited more than one peer. Similar studies conducted with youth samples have reported peer recruitment rates by seeds of 61.8% and 66.7%, although these studies had fewer initial seeds, higher incentives, and capped referrals at three per participant. Helms et al [11] highlighted that more initial seeds tended to result in less successfully recruited peers, possibly due to fewer resources being allocated to engage seeds or seek out seeds with a larger social network. This may explain the low rates of recruitment in our study, where seeds were recruited online (via a research panel for students or social media advertisements) and informed of the study requirements at the beginning of the survey rather than directly through research staff. A drawback of offering monetary incentives to participants is the increased potential of recruiting those who are not inherently engaged with the topic and therefore not motivated to refer their peers [10]. Since most participants were recruited from an online panel of students (Student Edge) who are used to completing short surveys in exchange for small payments, reaching out to peers to complete the survey—without the guarantee that they would finish it and thus receive extra payment—might not have been very appealing. This may also explain the finding from exploratory analyses that seeds recruited through social media were more likely to recruit their peers than those recruited through Student Edge.

Although recruitment through Student Edge allowed us to quickly recruit the remainder of our sample, it did not result in effective peer recruitment. Few WebRDS studies in the literature have used research panels to recruit peers. Stein et al [28] sampled seeds from two internet participatory surveillance panels comprising a community of adult volunteers who regularly report on health symptoms. While those in the panel with the chance to win a gift card were more likely to recruit peers, overall, both samples successfully recruited peers [28]. Notably, participants from that study likely had different motivations for engaging in the research (ie, interest in assisting in health surveillance) than our participants recruited through the Student Edge panel.

Although social media recruitment was more effective for recruiting peers, no seeds were recruited through Snapchat or TikTok, and only a small number through Instagram and Facebook. Currently, WebRDS studies have largely recruited young people and adults using Facebook, and there are few examples of researchers using any platforms other than Meta (Instagram and Facebook) [12,14,29]. Considering the key differences between social media platforms, video content should be tailored to the platform. For instance, our advertisements comprised short videos with colorful and dynamic text, yet TikTok recruitment videos typically feature an individual (ie, health professional or peer) introducing the research [30]. There is no clear formula for success in social media recruitment. There are many factors that contribute to recruitment success, such as ad style and content, advertising budget, the account that distributes the ad, and interactions between users and the ads [31]. While many studies have reported on successful recruitment and made recommendations such as use of colorful images, pop-culture memes, or bold text, other studies attempting to duplicate these methods fall short [31-34].

Consistent with other WebRDS research, successful and nonsuccessful seeds did not differ in gender, age [35,36], or education level [36]. Although some general population studies have reported that successful seeds are more likely to be younger (19-24 y), our age range was quite narrow (16‐18 y), suggesting that there may be no meaningful difference within these later teen years. Similarly, recruited peers did not differ in age, gender, or closeness of relationship with their referring seed, relative to nonrecruited peers. To our knowledge, no studies have directly examined this, although many have reported on homophily (the tendency for seeds to recruit peers with similar characteristics) present within samples [10]. Theoretically, it would be useful to understand relationship factors driving peers to engage in research (ie, if closeness of relationship was predictive of engagement, we could encourage seeds to only target these relationships). Nonetheless, most referred peers in our study had very (40.2%) or extremely (28.5%) close connections, suggesting that seeds are more inclined to recruit closer friends anyway. Overall, these findings emphasize the importance of focusing on financial reimbursement and recruitment source when attempting to recruit adolescent peer networks.

### Strengths and Limitations

Our study compared the effectiveness of two reimbursement strategies in a WebRDS study with adolescents aged 16 to 18 years in an RCT. We experimented using social media advertisements and a student panel to recruit young people, expanding beyond school-based relationships. The use of two-factor authentication allowed us to largely address the issue of fraudulent responses commonly reported in the WebRDS literature [10]. Nonetheless, we encountered other methodological issues, namely around the effectiveness of recruitment sources and recruitment of peers. Issues recruiting young people through social media resulted in a delay in recruitment while advertisements were revised. As a result, recruitment took place in two phases over a 2-year period. This does not appear to have significantly impacted the nature of the sample, as there were no key demographic differences between the two recruitment phases. A further deviation from protocol occurred because we could not reach a sufficient sample size from each recruitment source, and most participants were recruited through a market research panel. We have previously found that participants recruited through survey panels demonstrated greater attrition in a longitudinal study compared to those recruited through social media [37]. Another study showed that a research panel was significantly more representative than a sample recruited via social media [38]. It is recommended that future studies invest sufficient resources into creating advertising content that is engaging and relevant for specific social media platforms.

We were able to recruit sufficient seeds into each group, but the peer sample was smaller than anticipated based on previous literature. While the sample was sufficient to answer the primary research question, showing that scaled incentives are more effective than fixed incentives for participant recruitment, the smaller than anticipated number of peers recruited in both study arms raises questions about whether further methodological adjustments are needed to maximize peer recruitment. We found that those recruited through social media were more likely to refer peers than those recruited through the Student Edge panel. Further research is warranted to determine why that may be the case. As a result, the confidence intervals for exploratory analyses were wide. As such, results from secondary analyses should be interpreted with caution.

Notably, seven peers were unable to be linked with their seed using their codename, initials, and their seed’s ID number, highlighting a limitation of relying on manual linking methods. Due to the higher attrition rate post unblinding in the fixed intervention group, it is possible that participants stopped the survey when advised of their intervention group, which may overestimate the effectiveness of the intervention.

Finally, this study was conducted with a specific population of adolescents obtained through convenience sampling; hence, the sample is not representative of the population, and findings may not translate to other age groups and samples. Compared to Australian census data, our sample included a higher proportion identifying as lesbian, gay, bisexual, transgender, queer, intersex, asexual, and other identities (34% vs 10%), speaking a language other than English at home (37% vs 23%), identifying as Aboriginal and Torres Strait Islander (3.2% vs 1.9%), and attending government schools (65% vs 59%) [39].

### Conclusions

The present findings highlight important considerations for using WebRDS methodology to recruit adolescent peer networks. Specifically, adolescents are more motivated to recruit their peers to complete a survey when incentivized for each additional peer, although this does not guarantee a large peer network. Participants recruited through Instagram and Facebook were more likely to recruit their peers than those from the student panel, possibly due to different motivations for research involvement. However, peer networks remained small across both recruitment methods. Further research is warranted to understand how to best recruit adolescent peer networks outside of the school setting using financial reimbursements and different recruitment sources. With more time and cost-effective web-based approaches to recruiting peers, longitudinal studies can be conducted to investigate the influence of social networks on key health behaviors over time.

## Supplementary material

10.2196/70223 Checklist 1 CONSORT-eHEALTH (V 1.6.1) checklist.

## Abbreviations

CONSORT-EHEALTH: Consolidated Standards of Reporting Trials of Electronic and Mobile Health Applications and Online Telehealth
GEE: generalized estimating equations
RCT: randomized controlled trial
RDS: respondent-driven sampling
WebRDS: web-based respondent-driven sampling
